# First Report of *Belonolaimus Longicaudatus* Infecting Soybean in Indiana

**DOI:** 10.2478/jofnem-2022-0034

**Published:** 2022-11-12

**Authors:** John Bonkowski, William T. Crow, Alemayehu Habteweld

**Affiliations:** 1 Plant and Pest Diagnostic Laboratory, Purdue University, West Lafayette, IN 47907 US; 2 Entomology and Nematology Department, University of Florida, Gainesville, FL 32611 US

**Keywords:** *Belonolaimus longicaudatus*, detection, diagnosis, *Glycine max*, Indiana, soybean

## Abstract

Soybeans (*Glycine max*) are an important crop for Indiana, playing a major role in the state’s economy. In June 2021, symptomatic soybean plants were submitted to Purdue University’s Plant and Pest Diagnostic Laboratory for diagnosis. Sting nematodes were observed on the surface of the washed roots using stereo- and brightfield compound microscopy. A total of 76 sting nematodes per 100 cm^3^ soil were recovered from a composite soil sample. Morphological features and measurements of adult females and males of the sting nematode population were similar to those described for *Belonolaimus longicaudatus*. Molecular analysis confirmed the morphological identification using D2-D3 expansion segment of the 28S large subunit ribosomal DNA and internal transcribed spacer (ITS) regions. The consensus sequences were submitted to the National Center for Biotechnology Information (NCBI) database with accession numbers OM632679 and OM632681 for the D2-D3 and ITS regions, respectively. Parasitism of the sting nematode population to soybean plants was confirmed as its average population increased from 27 to 40 nematodes per pot 4 wk after inoculation under greenhouse conditions. To the best of our knowledge, this represents the first report of the sting nematode *B. longicaudatus* in Indiana.

*Belonolaimus longicaudatus* is among the most destructive plant-parasitic nematodes for a wide range of plants, including agronomic crops, ornamentals, forages, vegetables, turfgrasses, and trees (Duncan *et al*., 1996; Cid del Prado Vera and Subbotin, 2012). In addition to direct root damage, *B. longicaudatus* predisposes plants to stress caused by adverse conditions such as drought and heat, which could lead to poor yield and quality (Lucas, 1982). In June 2021, soybean (*Glycine max*) plants grown in approximately one-half of an acre within a farmer’s field located in Knox County, Indiana, were showing general above-ground stunting in patches within the field, foliar symptoms suggestive of micronutrient deficiency (Fig. 1A), and roots stunted, discolored, and lacking secondary branching (Fig. 1B). Symptomatic plants were submitted to the Purdue University’s Plant and Pest Diagnostic Laboratory for diagnosis, where sting nematodes were observed on the exterior of washed roots using stereo- and brighfield compound microscopy. A soil sample was submitted to Michigan State University’s Plant and Pest Diagnostic Laboratory’s Nematode Assay Service for evaluation. The sample was extracted using sugar-centrifugation method (Jenkins, 1964), yielding 76 sting (*Belonolaimus* sp.), 86 lance (*Hoplolaimus* sp.), four dagger (*Xiphinema* sp.), and 80 cyst (*Heterodera* sp.) nematodes per 100 cm^3^ of soil. Observation of morphological and morphometric characters of *Belonolaimus* females and males revealed that the population was representative of *B. longicaudatus* by having females with an offset lip region, a thin stylet >100 microns with rounded stylet knob, esophageal glands overlapping the intestine, and a hemispherical tail (Rau, 1958). Measurements (mean, range) of females (n=10) included body length 2,087 (2,042–2186) μm, vulva at 50% (48%– 52.2%), stylet length 125 (117–135) μm, and tail length 120 (110–135) μm. Measurements of males (n=6) included body length 1,761 (1,687–1,912) μm, stylet length 122 (117–128) μm, spicule length 47 (43–49) μm, and tail length 119.6 (110–129) μm. Molecular analysis was performed at the University of Florida’s Nematode Assay Laboratory to confirm the morphological identification by targeting the D2-D3 expansion segment of 28S rDNA and the internal transcribed spacer (ITS) regions using D2A and D3B, and AB28F and TW81R primer sets, respectively. Forward and reverse sequences of each individual primer set were subjected to alignment, and the complementary sequences were assembled into a consensus sequence. Using the National Center for Biotechnology Information (NCBI) (ncbi.nlm.nih. gov) BLASTn tool, both the D2-D3 and ITS regions showed 99% identity to those of *B. longicaudatus*. The accession numbers were submitted to the NCBI database as OM632679 and OM632681 for D2D3 and ITS regions, respectively. After confirmation of the nematode species, a test was conducted in the greenhouse to assess the pathogenicity and parasitism of the nematode population on soybean. Four soybean seedlings were grown in 0.5-l pots filled with autoclaved sand for and inoculated with 27 sting nematodes from a second soil sample collected from the original sampling location. Four uninoculated seedlings were used as controls. Greenhouse temperatures varied from 24°C to 26°C. Four weeks postinoculation, the inoculated plants were showing general stunting and subtle interveinal chlorosis; roots were severely stunted and showed brown lesions along their length. Uninoculated control plants appeared healthy, and the root system was white and filled the pot. On average, 40 nematodes were extracted from each pot using the decanting–sieving method (Ferris, 2019), indicating a reproduction factor (RF) of 1.43. It has been observed that pathogenicity studies involving sting nematode can yield low RF due to the significant damage they are capable of causing to the host (W. T. Crow, pers. comm.).

**Figure 1:**
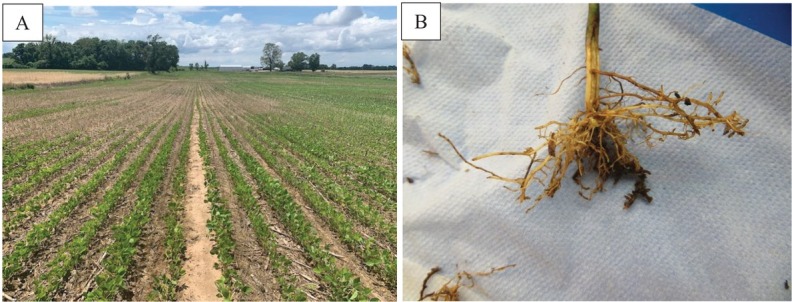
(A) A patchy appearance in the grower’s field where the sting nematode (*Belonolaimus longicaudatus*) was detected; (B) symptoms of root damage on plants uprooted from the patchy area.

Sting nematode is known to be a damaging pest to soybean (Barker *et al*., 1998). The fields where sting nematodes are found are used to grow agronomic crops in rotation using wheat, corn, soybean, and melon; grassy weeds are also present in the field. *Belonolaimus longicaudatus* is found predominantly in the sandy coastal areas of southeastern United States, where soils consist of >80% sand (Robbins and Barker, 1974; Crow, 2018). It is also common in the sandy regions along the Gulf of Mexico and the Atlantic coasts from Texas to Virginia and the sandy areas inland (Crow, 2018; Habteweld *et al*., 2021). However, sting nematode has not been reported from Indiana previously. To the best of our knowledge, this represents the first report of the sting nematode (*B. longicaudatus*) in Indiana and expands its known geographic range.

## References

[j_jofnem-2022-0034_ref_001] Barker K, Pederson, G., Windham G., Bartels J. (1998). Plant and nematode interactions, volume 36. Madison.

[j_jofnem-2022-0034_ref_002] Cid del Prado Vera I., Subbotin S. A. (2012). Belonolaimus maluceroi sp. n. (Tylenchida: Belonolaimidae) from a tropical forest in Mexico and key to the species of Belonolaimus. Nematropica.

[j_jofnem-2022-0034_ref_003] Crow W. T. (2018). Sting Nematode, Belonolaimus longicaudatus Rau (Nematoda: Secernentea: Tylenchida: Tylenchina: Belonolaimidae: Belonolaiminae).

[j_jofnem-2022-0034_ref_004] Duncan L. W., Noling J. W., Inserra R. N., Dunn D. (1996). Spatial patterns of Belonolaimus spp. among and within citrus orchards on Florida’s central ridge. Journal of Nematology.

[j_jofnem-2022-0034_ref_005] Ferris H. (2019). The Nematode-Plant Expert Information System (Nemaplex).

[j_jofnem-2022-0034_ref_006] Habteweld A., Mendes M. L., Inserra R. N., Crow W. T. (2021). Phylogentic relationship of some Belonolaimus longicaudatus populations associated with turfgrasses in the southeastern USA. Nematropica.

[j_jofnem-2022-0034_ref_007] Jenkins W. (1964). A rapid centrifugal-flotation technique for separating nematodes from soil. Plant Disease Reporter.

[j_jofnem-2022-0034_ref_008] Lucas L. T. (1982). Population dynamics of Belonolaimus longicaudatus and Criconemella ornata and growth response of bermudagrass and over seeded grasses on golf greens following treatments with nematicides. Journal of Nematology.

[j_jofnem-2022-0034_ref_009] Rau G. (1958). A new species of sting nematode. Proceedings of the Helminthological Society of Washington.

[j_jofnem-2022-0034_ref_010] Robbins R. T., Barker K. R. (1974). The effect of soil type, particle size, temperature, and moisture on reproduction of Belonolaimus longicaudatus. Journal of Nematology.

